# Effects of a teaching mode combining SimBaby with standardized patients on medical students’ attitudes toward communication skills

**DOI:** 10.1186/s12909-022-03869-8

**Published:** 2022-11-30

**Authors:** Ji-Dong Tian, Fei-Feng Wu, Chuan Wen

**Affiliations:** Department of Pediatrics, The Second Xiangya Hospital, Central South University, Changsha, 410011 China

**Keywords:** Simulation-based medical education, Standardized patient, Communication skills, Clinical teaching, SimBaby

## Abstract

**Objective:**

To evaluate the effect of a teaching mode combining SimBaby with standardized patients (SP) on medical students’ attitudes toward communication skills (CS).

**Methods:**

Forty 8-year medical program students majoring in clinical medicine were randomly divided into the SimBaby group (*n* = 20) and the SP + SimBaby group (*n* = 20). The Communication Skills Attitude Scale (CSAS) was used to evaluate medical students’ attitudes toward CS learning.

**Results:**

In the SimBaby and SP + SimBaby groups, there were no statistically significant differences in the Positive Attitude Subscale (PAS) and Negative Attitude Subscale (NAS) scores between males and females (*p* > 0.05). Compared to the SimBaby group, the SP + SimBaby group showed statistically significant differences in PAS, NAS, and the two dimensions of importance in medical context and learning (*p* < 0.05). There were no statistically significant differences between groups in the dimensions of excusing and overconfidence (*p* > 0.05).

**Conclusion:**

Compared with SimBaby alone, the SP + SimBaby teaching mode can improve medical students’ attitude toward CS learning, suggesting that the organic integration of multiple simulation-based medical teaching methods plays an important role in the acquisition of CS.

In 1993, the General Medical Council (GMC) proposed that medical students should have developed good communication skills (CS) by the end of their undergraduate education [1]. In 2002, the Institute for International Medical Education (IIME) proposed CS as one of the most basic requirements (other requirements including clinical skills, critical thinking, information management, etc.) in medical education worldwide, since doctor-patient CS is an important part of medical personnel’s practice skills [2]. In fact, health care communication is a critical component of medicine. Effective communication may promote harmonious doctor-patient relationships and improve patient outcomes such as satisfaction and adherence [3]. In another hand, ineffective doctor–patient communication is problematic as it can lead to nonadherence and other health-related issues. It is estimated that 35% to 70% of medicolegal actions result from poor delivery of information, failure to understand patient and family perspectives, etc., all of which can be avoided through good communication [4]. Therefore, communication is critical in medicine, and particularly in pediatrics. Pediatrics has a higher risk of medical conflicts and more difficulty in doctor-patient communication. Major sources of communication difficulty in pediatric medical visits have been identified, possibly including: the triadic nature of pediatric patient interactions would require more time since the child’s preferences and values should be solicited in addition to that of the parents; limitations in children's abilities to understand information and physicians' ability to judge what can be understood by children of different ages and development; patient and caregiver failure to express major concerns and worries [5–7]. Above all, programmatic or curricular emphasis on building communication skills is urged in pediatric service or training.

In order to ensure effective communication "behavior", medical education should include and integrate the "attitude" theme of medical students’ learning communication skills. In 2002, Rees et al. developed the CSAS, which can reliably identify medical students’ attitudes toward CS learning, asserting that attitudes toward CS learning are related to medical students’ demographics (such as gender and age) and education-related characteristics [8, 9]. The CSAS has good reliability and validity, and scholars in many countries and regions (such as Norway, South Korea, the United States and China) have adopted it to understand and evaluate the attitude of medical students toward doctor-patient CS [10–13].

Clinical medicine is a highly practical applied discipline. As medical models have changed, simulation-based medical education (SBME) has, consequently, become a new development trend that has been applied and promoted in actual clinical medicine teaching [14]. The current simulation tools include human patient simulators (such as SimBaby and SimMan), task trainers (such as a tracheal intubation trainer), standardized patients (SP), virtual reality simulation, and hybrid simulations that involve the simultaneous use of 2 or more methods of simulations [15]. SimBaby is a multimedia baby simulator that can realistically simulate some typical pediatric cases and can be used to train and assess medical students’ clinical diagnosis and operation skills [16]. Our teaching experience showed that directly examination, diagnosis and management of SimBaby by medical students without prior inquiry was not conducive to the training of CS. SPs, can perform the case characteristics of different ages according to the needs of the role, have been trained and used in clinical teaching and assessment [17, 18]. Because age limits the cooperation and safety during the simulation, it is very difficult to train infant SPs for physical examination and operation practice. Since each type of simulation tool has its advantages and limitations, in terms of some difficulty and clinical variation, especially in pediatric medical teaching, it is sometimes necessary to use hybrid simulations. For example, our previous study showed that hybrid simulation combining SimBaby and SP (act as the parents of patients) can improve medical students’ medical knowledge and learning enthusiasm [19]. Anna et al. designed a hybrid simulation model for pediatric and adolescent gynecology examination teaching, which was feasible and greatly accepted by the trainee and improved learning attitudes [20, 21]. However, the effect of hybrid simulation model on medical students’ CS learning attitude is still poorly studied. This study used the CSAS to evaluate the effect of the teaching mode combining SimBaby with SPs on medical students’ attitudes toward CS learning.

## Research subjects and methods

### Subjects and groups

Forty 8-year medical students majoring in clinical medicine (all the students were from the same grade and there were no differences in their courses and trainings of clinical practices, simulation education, and pediatric education) were randomly divided into the SimBaby group (*n* = 20), which included eleven males and nine females, and the SP + SimBaby group (*n* = 20), which included twelve males and eight females. In the SimBaby group, the teacher directly provided the patient’s medical history verbally to the students, after which the students operated on the SimBaby. In the SP + SimBaby group, SPs were employed as parents of patients, whose task were to provide medical history and informed consent during the medical process. After taking the detailed history from the SP, the students operated on SimBaby under comprehensive assessment and judgment. For the students in the two groups, the six roles of emergency physician, intern, resident physician, superior physician, nurse, and recorder were randomly determined by lottery, and medical teams were formed to complete the SimBaby operation. This study was approved by the Ethics Committee of the Second Xiangya Hospital of Central South University. The informed consent was obtained from all subjects.

### SP preparation and training

Adults with common medical knowledge were selected to be SPs, and the necessary medical history inquiry items for infants with acute bronchopneumonia combined with heart failure and cardiac arrest were developed. The SP was provided with the script about the disease condition and had to understand and be familiar with the script under the guidance of the training instructor. The SP was required to be proficient in memorizing the correct expressions of each item, to answer questions only when asked, to follow the script strictly, and to communicate with the medical student as a parent to provide the student with a detailed medical history for the subsequent SimBaby operation and participate in postoperative discussion and feedback.

### SimBaby implementation method

SimBaby preparation before operation: ①Scenario description: This case involved an 6 months old infant with heart failure and cardiac arrest due to severe acute bronchopneumonia. The mother of the child (SP) complained to the physicians that the child suddenly appeared cyanotic and did not wake up 5 min ago. The subjects were asked to elicit a history from the SP and to perform a physical examination to determine that the infant was cardiac arrest. The participants were required to treat the SimBaby with appropriate interventions for superior life support. After successful CPR, the physician needs to take a detailed history from the family and communicate the next steps in treatment. The teacher preselected a variety of treatment results in the SimBaby “program editor”. SimBaby can simulate relevant signs and present different prognoses based on the different treatments used by students according to the framework structure diagram. ②SimBaby training before operation. The teacher introduced the main functions of SimBaby to the students, demonstrated various clinical symptoms and signs and conducted basic life support and advanced life support training on SimBaby. Then, the teacher introduced the responsibilities of each role in the medical team to align the teaching activities more closely with the actual clinical work situation. However, the members of the medical team could not discuss the diagnosis and operation practice among themselves, and team members could only aid in various activities.

SimBaby operation. ①The medical students in the SimBaby group received the patient’s medical history verbally from the teacher. In the SP + SimBaby group, after taking the medical history from the SP, the internist or emergency physician of each team performed a physical examination. During the training, a teacher controlled the SimBaby through a computer in the control room, adjusting its symptoms and signs to ensure that the SimBaby followed the plan; another teacher observed and recorded students’ operations in the operation room. In the operation room, the students performed timely and relevant treatment for SimBaby according to its changes in condition. ②After the simulation, all teachers, SPs and medical students participated in the discussion and feedback.

### Questionnaire survey

#### Questionnaire design

Rees et al. developed and used the English version of the Communication Skills and Attitudes Scale (CSAS) to measure medical students’ attitudes toward CS. This scale is the most widely used tool to assess the attitude of medical students toward CS learning [8, 9]. In this study, two professional translators (one medical professional translator and one nonmedical professional translator) independently completed a Chinese translation of the English version of the CSAS. After translators and the main researchers discussed the translated content and intended meaning, a final consensus on the Chinese version of the CSAS was reached. Then, additional medical students were invited to perform a pretest to examine whether the subjects could understand the scale items, following which corresponding revisions were made. The final Chinese version of the CSAS was then complete.

#### Survey content

The CSAS consists of both positive and negative statements, with a total of 26 items. Negative and positive statements are presented in an arbitrary order, thus forming two subscales: the Positive Attitude Scale (PAS; a total of 13 statements) and the Negative Attitude Subscale (NAS; a total of 13 statements). A five-point Likert scale is used, i.e., there are five choices at the end of each statement that represent scores from 1 to 5: “strongly disagree”, “disagree”, “neutral”, “agree” and “strongly agree”. Therefore, the scores of the two subscales range from 13 to 65 points, where higher scores indicate stronger positive or negative attitudes toward CS learning. In the questionnaire survey, participants are asked to score each item from 1 to 5. In this study, the PAS score can be obtained by summing the scores of CSAS items 4, 5, 7, 9, 10, 12, 14, 16, 18, 21, 23, 25 and the inverse score of item 22, and NAS score can be obtained by summing the scores of CSAS items 1, 2, 3, 6, 8, 11, 13, 15, 17, 19, 20, 24, and 26. In this study, PAS and NAS were scored separately according to medical students’ gender.

The 26 items in the CSAS questionnaire can be classified into the following four dimensions: importance in medical context, with 11 items (1, 4, 5, 9, 10, 14, 16, 19, 21, 23, and 25); excusing, with six items (2, 6, 8, 15, 18, and 26); learning, with six items (7, 11, 12, 13, 17, and 24); and overconfidence, with three items (3, 20, and 22). The importance in medical context dimension represents medical students’ attitudes toward respecting patients and colleagues, recognizing patients’ rights, and teamwork; the excusing dimension represents medical students’ attitude toward the reasons for refusal to participate in CS training courses; the learning dimension represents students’ attitudes toward learning; and the overconfidence dimension represents the learners’ low demand for CS learning [22]. In this study, the above four dimensions were scored and analyzed.

#### Data collection

After the two groups of medical students completed the SimBaby and SP + SimBaby courses, students were invited to participate voluntarily in the CSAS questionnaire. They were informed of the anonymous data analysis. After brief instructions were provided, the questionnaire was distributed to students by a teacher, who did not mention the purpose of this study. To ensure survey accuracy, medical students were required to complete the questionnaire independently.

### Statistical analysis

SPSS software (version 21.0) was used to analyze the data. Measurement data were expressed as the mean ± standard deviation (SD). The student *t* test was performed for group comparison. *p* < 0.05 (^*^) was regarded as statistically statistically significant.

## Results

### General information

All students participating in the survey were selected based on the same national standard entrance examination (i.e., college entrance examination) and entered medical school in the same year. There were no differences in age, curriculum, and clinical training among medical students. A total of 40 questionnaires were distributed in this study, and 40 were recovered, with an effective recovery rate of 100%.

### The effect of gender on PAS and NAS

In the SimBaby and SP + SimBaby groups, there were no significant differences in PAS and NAS between male and female students in each group (*p* > 0.05), which indicates that there is no gender difference in medical students’ attitude toward CS learning (Table 1).

**Table 1:** Effect of medical students’ gender on PAS and NAS

Scores	SimBaby			SP + SimBaby		
	Male (*n* = 11)	Female (*n* = 9)	*P*	Male (*n* = 12)	Female (*n* = 8)	*P*
PAS	30.12 ± 2.76	32.78 ± 1.98	0.782	45.33 ± 1.77	47.11 ± 1.05	0.564
NAS	19.73 ± 1.69	20.45 ± 2.33	0.436	17.67 ± 2.12	16.55 ± 2.67	0.672

*PAS* Positive Attitude Subscale, *NAS* Negative Attitude Subscale, *SP* Standardized Patient

### The effects of the two teaching modes on various CSAS scores

Compared to the SimBaby group, the SP + SimBaby group showed statistically significant differences in PAS, NAS, and the two dimensions of importance in medical context and learning (*p* < 0.05), which indicates that the integrated teaching mode SP + SimBaby, compared with SimBaby alone, can improve medical students’ enthusiasm for CS learning, reduce their negative attitude toward CS learning, and help them realize the importance of CS learning in the medical context. There were no statistically significant differences in the dimensions of excusing and overconfidence between the SP + SimBaby and SimBaby groups (*p* > 0.05), which suggests that there are no differences in the reasons for medical students to refuse to participate in CS training courses nor their low demand for CS learning and development between the two teaching modes (Table 2).

**Table 2:** The effects of two teaching modes, SimBaby and SP + SimBaby, on various CSAS scores

	**PAS**	**NAS**	**Importance in** **medical context**	**Excusing**	**Learning**	**Overconfidence**
SimBaby **(*n* = 20)**	39.33 ± 1.63	22.55 ± 1.79	41.67 ± 2.16	17.67 ± 2.53	20.56 ± 1.47	8.11 ± 1.23
SP + SimBaby **(*n* = 20)** *P* value	43.44 ± 1.62 0.017	18.27 ± 2.19 0.008	48.33 ± 3.22 0.021	16.89 ± 2.17 0.785	25.36 ± 2.38 0.037	7.76 ± 1.35 1.064

*PAS* Positive Attitude Subscale, *NAS* Negative Attitude Subscale, *SP* Standardized Patient

## Discussion

Modern medical education needs not only to improve scientific knowledge but also to promote medical students’ medical humanistic literacy, of which CS is an important element. Medical students have been increasingly trained and assessed in CS worldwide [23–25]. Communication evaluation includes two aspects, namely, the evaluation of communication ability and learning attitude. For the former, there are relatively systematic studies, with extensive evaluation content, mature evaluation tools and diverse evaluation methods; however, there are few studies on attitudes toward CS learning. The CSAS, with good reliability and validity, is currently the most widely used tool for evaluating medical students' attitudes towards CS learning. Many countries and regions (such as Norway, South Korea, the United States and China) used PAS and NAS to evaluate the learning attitude of medical students' communication skills, founding that there are differences formed by corresponding national demographics [10–13]. Previous tudies have shown that gender affects medical students’ attitudes toward CS learning, manifested as higher PAS scores and lower NAS scores for female students than male students [9, 13, 26]. However, in the two teaching modes in this study, there was no gender difference in the learning attitude toward CS, which may be related to the small sample size.

Pediatric severe pneumonia complicated by heart failure and cardiac arrest is a classic clinical case that requires rapid recognition and resuscitation of the patient by the physician. Because of the severity of the illness, it is not possible to use real cases in medical teaching. SimBaby can be a good alternative by simulating the relevant signs and showing different outcome responses depending on the treatment. Medical students' operational capability (such as cardiopulmonary resuscitation) can be satisfactorily trained using SimBaby. However, in the actual practice of pediatric medicine, the patient's guardian (usually the parent) always plays an important role. The physician needs to communicate with the guardian continuously to obtain a medical history, inform them of the condition, obtain consent for treatment, etc. This process is known as doctor-patient communication. We chose SP to act as the patient's (SimBaby) parent, which more completely and realistically simulates the entire medical process.

Participants in this study did not take any CS training courses and lectures prior to enrollment. Compared with SimBaby alone, SP + SimBaby can increase medical students’ positive attitudes and decrease their negative attitudes toward CS learning, as reflected in higher PAS and lower NAS scores. Therefore, the hybrid simulation mode SP + SimBaby can improve medical students’ attitudes toward CS learning, suggesting that the simultaneous use of multiple simulations could play an important role in CS learning. This may be because in the process of communicating with SP, students were more likely aware of inadequacies in CS [27]. Studies have shown that integrating simulation with art-based teaching can improve the attitude of oncology residents to CS learning [22]. Therefore, the integration of several teaching methods is of great significance to doctors’ CS training.

Next, the effect of SP + SimBaby model on the four dimensions of CSAS was analyzed. First, the dimension of importance in medical context represents medical students’ attitudes toward respecting patients and colleagues, recognizing patients’ rights, and teamwork, and represents an important element of positive attitude in the CSAS. SimBaby is a highly realistic high-fidelity patient simulator, but it nonetheless has limits for students’ CS training. In this study, SPs were integrated into SimBaby teaching, which greatly improved the dimension of importance in medical context (48.33 ± 3.22 vs 41.67 ± 2.16, *p* = 0.021). In addition, students in the SP + SimBaby group scored higher on the learning dimension, implying a more positive attitude toward learning (25.36 ± 2.38 vs 20.56 ± 1.47, *p* = 0.037). Unlike obtaining a complete and correct medical history directly, students in the SP + SimBaby group needed to communicate with the SPs to obtain a medical history and explain the patient's condition and treatment. During this process, students were more likely to discover their own medical knowledge deficiencies. And then their positive attitudes toward CS learning were stimulated, which less common in simple SimBaby teaching. Furthermore, studies have shown that teaching creates negative learning attitudes [28, 29], the degree of negativity is affected by the different courses in medical colleges [30, 31], and students’ negative attitude toward learning is more obvious among senior students and senior doctors [32]. In this study, there were no differences in the scores on the two dimensions of excusing and overconfidence between the two teaching modes, which may be because the two groups of students are young (e.g., their enthusiasm for learning is high) and have not taken CS training courses and lectures. The relationships between the two dimensions of excusing and overconfidence and time need to be further explored in a longitudinal study.

Attitude can drive behavior. If a person’s attitude can be changed, his or her behavior may be changed too [32]. In this study, compared to SimBaby alone, the bybrid mode SP + SimBaby can improve medical students’ attitude toward CS learning, proving that the appropriate combination of simulation tools can play an active role in CS learning. For example, in the teaching of the reproductive system examination, we can train older children as SPs, and the physical examination can be operated in the task trainers. Furthermore, virtual reality simulation, such as virtual pediatrics SPs using advanced computer technology, provides great promise for clinical skills education. However, this study has the following limitations. This study is a cross-sectional study; therefore, longitudinal studies on medical students’ attitudes toward CS learning are needed to determine whether this change can be maintained over time, and qualitative studies are also needed to explain the change in medical students’ attitudes toward CS learning. This study is a single-center study with a small sample size. Therefore, for other medical schools or research centers, the external validity and universality of the results of this study need to be further studied.

## Data Availability

All data generated or analysed during this study are included in this published article.

## Abbreviations

SP: Standardized patients
CS: Communication skills
CSAS: Communication Skills Attitude Scale
PAS: Positive Attitude Subscale
NAS: Negative Attitude Subscale
GMC: General Medical Council
IIME: The Institute for International Medical Education
SBME: Simulation-based medical education
SD: Standard deviation
